# Study of Nitrogen Removal Performance When Treating Low Carbon Sewage Using External Solid Carbon Sources in SBBR Systems

**DOI:** 10.1155/2017/3691819

**Published:** 2017-12-14

**Authors:** Li-qiu Zhang, Pei-fen He, Dan Wu, Shu-geng Li, Yi-liang Huang, You-wen Huang, Deng-min Wang

**Affiliations:** ^1^School of Civil Engineering, Guangzhou University, Guangzhou 510006, China; ^2^Key Laboratory for Water Quality and Conservation of the Pearl River Delta, Ministry of Education, Guangzhou University, Guangzhou 510006, China; ^3^School of Environmental Science and Engineering, Guangzhou University, Guangzhou 510006, China

## Abstract

Based on low carbon wastewater as the research object and using corncob as an external solid carbon source, the performance of corncob organic matter was assessed for its release potential, quantity of release, and safety of use. The effects of varying quantities of the solid carbon source on simultaneous nitrification and denitrification were investigated in a sequencing biofilm batch reactor (SBBR). Results show that the regularity of corncob as solid carbon source material was linear, with released concentrations of heavy metals being below the Chinese national standard limit values for heavy metals according to the surface water environment quality standards (I and II) (GB3838-2002). When temperatures were within 28~31°C, the dissolved oxygen level was 4.0 ± 0.2 mg/L and the pH conditions were within 7.5~8.0. The optimal quantity for corncob dosing was 5 g per 1.5 L of low carbon wastewater. Following treatment, the average effluent concentrations of NH_4_^+^-N and TN were 2.85 mg/L and 4.51 mg/L, respectively. The effluent concentration of NH_4_^+^-N, TN had reached the A level national standard of sewage treatment plant pollutant discharge standard (GB18918-2002).

## 1. Introduction

Recently, with the constant development of social economies and urbanization, significant pressures have been placed on our environment resources. An example of this is the discharge of wastewater and water pollutants into the aquatic environment, from both domestic, agricultural and industrial sources, posing a major threat to water resources and aquatic environment. In particular, the level of discharge of nitrogen containing sewage has increased rapidly, while the phenomenon of low carbon source wastewater is becoming an increasingly common issue, causing major problems in sewage treatment plants and causing the need for upgrading and retrofitting [1–3].

At present, sewage treatment plants generally use a liquid carbon source for denitrification, such as carbinol, acetic acid, or amylaceum, although some shortcomings exist with this method. It can be difficult to accurately control the quantities of carbon sources, with complicated operation procedures, and there is a risk of causing secondary organic pollution of effluent, which will greatly increase the cost of pretreatment systems [4–7]. Therefore, there is much current research focus on the optimization of denitrification technologies and understanding the role of solid carbon sources [8–12].

This study assesses synthetic low carbon sewage treatment, using corncob as an agricultural solid waste material providing an external solid carbon source. The release of carbon and heavy metals were quantified, allowing the safety and performance of corncob organic matter to be assessed. The effectiveness of varying quantities of solid carbon source in the removal of NH_4_^+^-N and TN was assessed during simultaneous nitrification and denitrification in a sequencing biofilm batch reactor (SBBR). And optimal quantity on simultaneous nitrification and denitrification in sequencing biofilm batch reactor (SBBR) were confirmed.

## 2. Materials and Methods

### 2.1. Reagents and Materials

The corncob utilized as an external carbon source was purchased from Guangzhou farmers market. Once returned to the laboratory, the corncob was washed with running water, cut into 1 cm^3^ segments, and dried in the oven at 80°C. Once cooled, corncob segments were placed on a drying container for preservation. To reduce variation, we utilized the same batch of corncob material for the duration of the experiment.

The reactor consisted of an organic glass column, with an internal column diameter of 14 cm, column height of 115 cm, containing eight biomembrane diaphragm filters, with an effective volume of 12 L. The synthetic sanitary low carbon wastewater was adopted in this experiment, containing starch, NH_4_Cl, KH_2_PO_4_, CaCl_2_, MgSO_4_·7H_2_O, FeSO_4_·7H_2_O. To obtain varying influent qualities, adjustments were made to the concentration of the main ingredients, starch and NH_4_Cl. During the film-forming period of the experiment, sufficient sources of organic substances and carbon were supplied to ensure enough nutrition and energy was available for the promotion of biomembrane formation, reproduction, and growth. The influent concentration of COD_Cr_ remained consistently within 250~300 mg/L, while the concentration of NH_4_^+^-N varied within 19.8~27.5 mg/L. Biomembranes were cultured 25 d until they remained attached, at which point the concentration of COD_Cr_ was reduced 90~120 mg/L and systems were allowed to continue acclimatizing 15 d.

### 2.2. Experiment Design

#### 2.2.1. Static Experiment to Assess Carbon and Heavy Metal Release from Solid Carbon Source Material

The corncob material utilized as a solid carbon source was weighted into 5 g, 10 g, 15 g, and 20 g portions, which were then divided into eight 2 L beakers, submerged in 1 L of deionized water, and sealed using a preservative film. Beakers were divided into separate groups for TOC and COD_Cr_ analysis. Samples were collected using an injector at 12 h, 24 h, 36 h, 48 h, 64 h, and 72 h, for the analysis of concentrations of TOC, TC, and COD_Cr_ in the water surrounding the immersed corncob. Analysis of the concentration of heavy metals in water surrounding the immersed corncob was performed at the end of the static experiment.

#### 2.2.2. Assessment of Optimum Dose of Solid Carbon Source Material

Parallel experiments were performed, with control levels for every factor simultaneously applied to ensure uniformity of conditions. Every period is 12 h; 12 L of wastewater was treated using the SBBR system containing eight biomembranes, resulting in 1.5 L of wastewater treated per biomembrane. Each cultured biomembrane was transferred to a 2 L beaker and submerged in 1.5 L of synthetic influent along with the corncob material at varying doses of 1.25 g, 3.75 g, 5 g, 7.5 g, and 10 g and the corncobs are replaced every 6 periods, when the characteristics of effluent are stable, showing the ending of sludge acclimatizing.

### 2.3. Analytical Measurements

The parameters measured during wastewater treatment were the concentration of COD_Cr_, NH_4_^+^-N, NO_2_^−^-N, NO_3_^−^-N, TN, pH, TOC/TC, and heavy metals and were measured according to the national standard methods [13]. The pH and DO were measured using pH probes (WTW-pH/Oxi340i, Germany).

## 3. Results and Discussion

### 3.1. Effectiveness of Corncob Material as a Carbon Source

**Figure 1:**
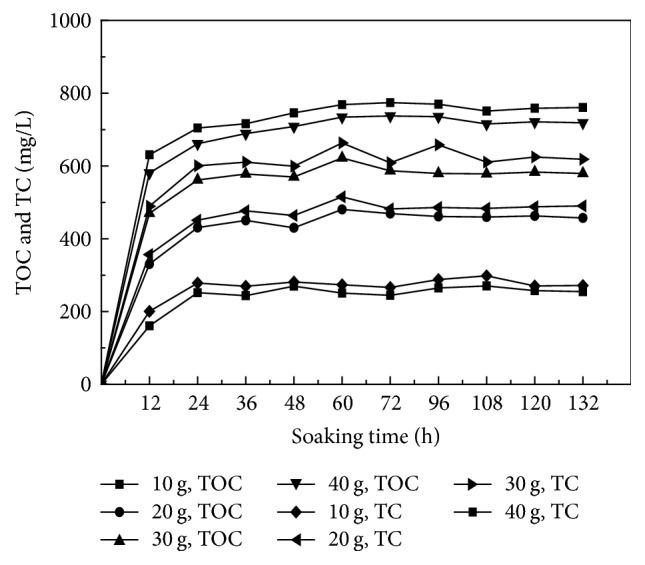
Level of release of TOC and TC from corncob at varying doses into surrounding deionized water.

**Figure 2:**
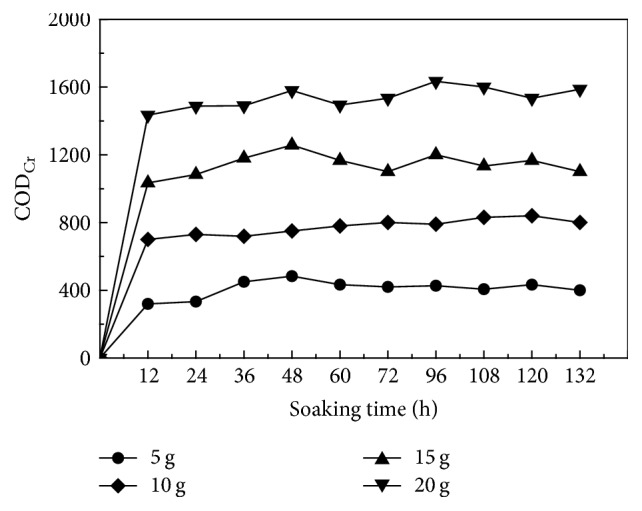
Level of release of COD_Cr_ from corncob at varying doses into surrounding deionized water.

The corncob solid carbon material was assessed for its level of carbon release at doses of 5 g, 10 g, 15 g, and 20 g, with concentrations assessed every 12 h, up to 132 h. Concentrations of TOC, TC, and COD_Cr_ were assessed in the water surrounding the immersed corncob, with results provided in Figures 1 and 2.

Figures 1 and 2 show that the concentrations of TOC and TC increased according to immersion time, to varying degrees depending on the dose of corncob material immersed in 1 L deionized water. After 24 h of immersion, TOC and TC concentrations were rapidly increased due to release from the corncob material, while, from 24 h to 96 h, concentrations fluctuated and then stabilized after 96 h. The concentrations of TOC and TC released from corncob material increased according to the quantity of corncob material applied, following the same immersion time. After the rate of release of carbon stabilized, a distinct linear relationship with time was observed. In water samples exposed to different quantities of corncob material, the ratio of TOC to TC was consistently over 90%, showing that corncob effectively releases high concentrations of organic carbon. In addition, corncob has the ability to provide an immediate carbon source, with a relatively stable rate of carbon release, suggesting that corncob may be a suitable material to apply as a solid carbon source in wastewater treatment.

During the initial 12 h, corncob was found to have a rapid release of high concentrations of COD_Cr_ and TOC under all scenarios assessed, although the rate of release was not consistent. The concentrations of COD_Cr_ increased according to immersion time, until 48 h when a steady state was maintained. The average concentration of COD_Cr_ released from corncob material was 406.9 mg/L released from 5 g corncob, 746.9 mg/L released from 10 g corncob, 1137.4 mg/L released from 15 g corncob, and 1503.4 mg/L released from 20 g corncob. In the initial 12 h, the rapid increase in concentrations of COD_Cr_ is due to water-soluble material on the surface of the corncob being degraded, with inner smaller molecules quickly dissolving into the surrounding media, increasing the effluent concentrations of COD_Cr_. After 48 h a steady state was observed as most available COD_Cr_ has been released via hydrolysis and the remaining corncob inner fiber is formed of material that is difficult to decompose, such as lignin. It is of note that after immersion for 48 h corncob material showed signs of ageing, with white regions appearing and a foul odor, potentially because the decomposition of corncob material was restrained.

By calculating the average level of release of COD_Cr_ during the linear period, from varying quantities of corncob, the average concentration of carbon released by 1 g corncob was 76.8 mg/L deionized water.

### 3.2. The Safety of Corncob Material as a Solid Carbon Source

It has been established that there are some elements released into surrounding water from corncob material, such as Ca, K, Mg, Na, Si, and P, among others, which are indispensable nutritional materials and essential constituents for microbes. Additionally, metal microelements are also released, which have an important role in microbial growth and reproduction. The enzyme active gene consists of inorganic mineral elements for adjusting the osmotic pressure in cells. It has been reported that metal ions released from carbon source materials into surrounding water during the process of denitrification can increase the fermentation activity of microbes and therefore increase the rate of denitrification [14]. Standard limit values (Table 1) exist for concentrations of metal ions in surface water (environment quality standard [15] (GB 3838-2002)), with any excess of these limits presenting a hazard to biofilm microbes and the natural environment. Therefore, when selecting a solid carbon source material, the safety and potential release of harmful substances must be considered. To assess the safety of corncob material as a solid carbon source, 6 kinds of typically regulated heavy metals were studied, including As, Cu, Cd, Cr, Pb, and Zn.

The results show that no Cd was detected in the corncob immersion water, while the concentrations of Cu, Cr, and Zn were lower than the II class limit value and the concentration of Pb was below the I class limit value. It is of note that the water flow and velocity applied were significantly far less than in actual water-treatment situations, in addition the large volume of reactors used in wastewater treatment mean that the concentration of metal ion would be diluted greatly. Therefore, based on these findings, we may assume that the use of corncob as an external solid carbon source does not result in harmful levels of release of heavy metals and is not likely to pose a risk to environmental safety.

### 3.3. Effects of Varying Quantities of Solid Carbon Source in an SBBR System Treating Low Carbon Sewage

**Figure 3:**
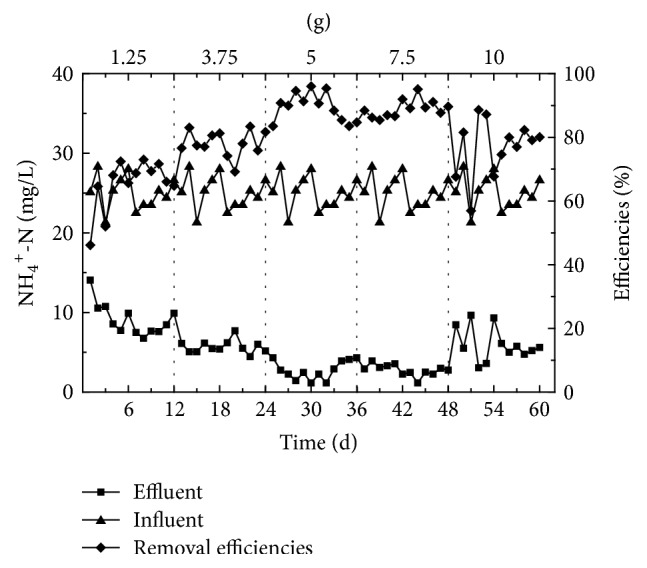
The degree of NH_4_^+^-N removal from synthetic wastewater following exposure to varying quantities of corncob.

**Figure 4:**
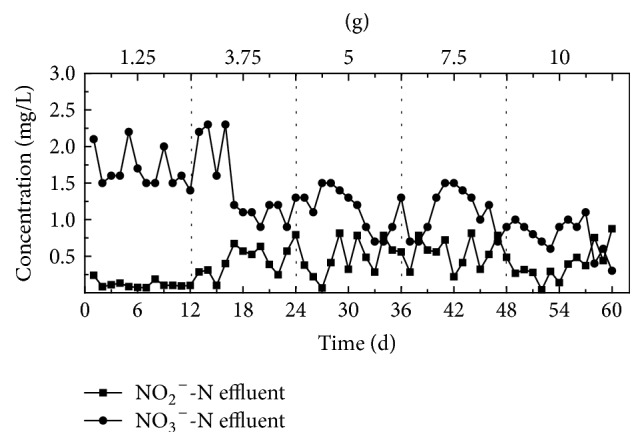
The degree of NO_2_^−^-N and NO_3_^−^-N removal from synthetic wastewater following exposure to varying quantities of corncob.

**Figure 5:**
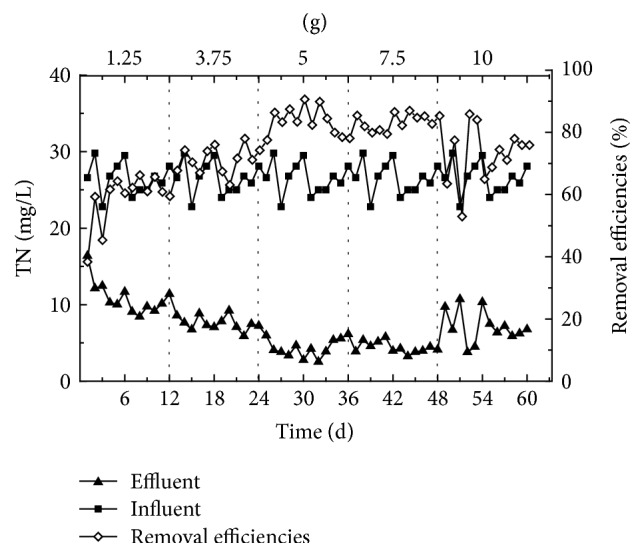
The degree of TN removal from synthetic wastewater following exposure to varying quantities of corncob.

The synthetic low carbon urban sewage was used in influent of these experiments. The influent concentration of COD_Cr_ remained consistently within 250~300 mg/L, while the concentration of NH_4_^+^-N varied within 19.8~27.5 mg/L. The concentration of TN was 21.5~29.6 mg/L, the temperature was 28~31°C, the DO was 4.0 ± 0.2 mg/L, and the pH value was at 7.5~8.0, The synthetic low carbon urban sewage was treated with varying quantities of corncob material (1.25 g, 3.75 g, 5 g, 7.5 g, and 10 g), while all other conditions remained unchanged. The water quality of synthetic effluent was assessed following steady operation for a period and the results of the continuous 12 periods are shown in Figures 3–5.

When the dose of corncob applied was within 1.25~5 g, the average removal ratio of NH_4_^+^-N increased according to increased quantity. When the applied dose of corncob was increased to 5 or 7.5 g, a stable level of removal NH_4_^+^-N was achieved, with an average removal ratio of 89% and 90%, respectively. Therefore, increased quantities of corncob material result in increasing the available carbon source and, consequently, an improved average removal rate of NH_4_^+^-N was observed. Conversely, when the dose of corncob applied was increased to 10 g, the average effluent concentration of NH_4_^+^-N increased, showing that the average removal ratio of NH_4_^+^-N was reduced. This phenomenon is likely due to the high concentration of COD_Cr_ causing a reduction in competition in nitrifying species following an extended period of operation, which is not effective in the removal of NH_4_^+^-N.

Figures 4 and 5 show the effectiveness of removing NO_2_^−^-N, NO_3_^−^-N, and TN from synthetic wastewater, under different corncob dose quantities.  Figure 4 shows that average effluent concentrations of NO_2_^−^-N and NO_3_^−^-N remained consistently low under all tested quantities of corncob and that the denitrification rate increased according to increased corncob quantity. The denitrifying bacteria is key heterotrophic and facultative anaerobes during the denitrification processes, which, under anaerobic conditions, utilize the carbon source, and NO_2_^−^-N and NO_3_^−^-N as electron donors to support denitrification processes [16–18]. Therefore, a close interaction exists between the concentration of the organic carbon source and the rate of denitrification in sewage, with the corncob material providing all organic carbon in this study, allowing the dosed quantity of corncob to be the determining factor for promoting the rate of denitrification. Due to average effluent concentration of NO_2_^−^-N and NO_3_^−^-N being consistently low, the overall removal rate of TN was dominated by changes in the removal rate of NH_4_^+^-N, when the dosed quantity of corncob was within 1.25~5 g, the average removal rate of TN increased according to carbon source quantity. However, when the dosed quantity of corncob was 5 g or 7.5 g, the effect of TN removal was more stable, with average removal rates of 83% and 84%, respectively. When the dosed quantity of corncob was increased to 10 g, the average removal rate of TN decreased slightly; this effect is likely due to excessive organic matter being released by corncob, causing the increase of denitrification rate but decrease of nitrification rate.

**Figure 6:**
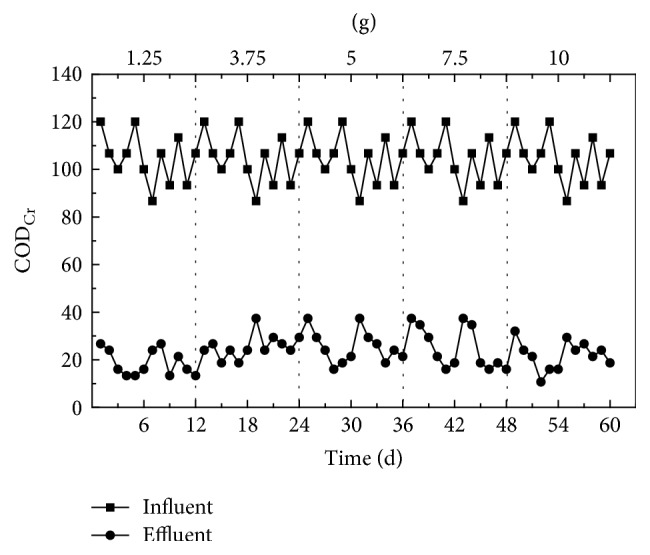
The change of COD_Cr_ concentrations in effluent following exposure to varying quantities of corncob solid carbon source material.

As shown in Figure 6, when the dosed quantity of corncob ranged from 1.25 to 10 g, the effluent concentration of COD_Cr_ remained largely stable below 30 mg/L, showing that no effect was observed on the removal rate of COD_Cr_ when the dosed quantity of corncob varied within 1.25~10 g.

Overall, corncob was found to be effective as an external solid carbon source for the treatment of low carbon wastewater in SBBR systems, with optimal effects being observed when the dosed quantity of corncob was within 5 g~7.5 g per 1.5 L of wastewater. The use of 5 g corncob resulted in an average effluent NH_4_^+^-N concentrations of 2.85 mg/L, an average NH_4_^+^-N removal rate of 89%, with average effluent TN concentrations of 4.51 mg/L, an average TN removal rate of 83%, and average effluent concentrations of NO_2_^−^-N and NO_3_^−^-N, of 1.16 and 0.48 mg/L, respectively. Following treatment, the quality of effluent reached the level of A standard in “urban sewage treatment plant pollutant discharge standard" (GB18918-2002).

## 4. Conclusions

Corncob was found to be an effective external solid carbon source, showing a consistent release of carbon source, with a rapid level of release in the first 12 h and a stable rate of carbon release after 24 h. This pattern of carbon release was observed at all tested dose quantities of corncob organic matter. In addition, no harmful levels of heavy metal ions were released from corncob, with surrounding water metal content being below the standard limit value.

When corncob was applied as an external solid carbon source to treat low carbon wastewater in an SBBR system, the best dosed quantity of corncob was 5 g per 1.5 L wastewater. The average effluent concentrations of NH_4_^+^-N and TN were reduced from over 19.8 mg/L and 21.5 mg/L to 2.85 mg/L and 4.51 mg/L, respectively. The average effluent concentrations of NO_2_^−^-N and NO_3_^−^-N were reduced to 1.16 mg/L and 0.48 mg/L, respectively. Therefore, the effluent water quality was treated to level A of the standard in urban standard of wastewater treatment plant pollutant discharge standard (GB18918-2002).

## Figures and Tables

**Table 1:** Standard limit values for heavy metals according to the surface water environment quality standards (I and II), with the concentration of heavy metals released from corncob material into surrounding water.

Metal species	The level of I (mg/L)	the level of II (mg/L)	corncob (mg/L)
Cu	0.01	1.0	0.023
Zn	0.05	1.0	0.134
As	0.05	0.05	0.006
(+6) Cd	0.001	0.005	n.d.
Cr	0.01	0.05	0.02
Pb	0.01	0.01	0.006

n.d. means undetected.
